# Supplementary material for: Test accuracy of polymerase chain reaction methods against conventional diagnostic techniques for Cutaneous Leishmaniasis (CL) in patients with clinical or epidemiological suspicion of CL: Systematic review and meta-analysis
Source: PLoS Negl Trop Dis. 2020 Jan 21;14(1):e0007981. doi: 10.1371/journal.pntd.0007981 (PMC6994169; doi:10.1371/journal.pntd.0007981)
Supplement: Attachment — Submitted filename: Response to Reviewers.pdf [file pntd.0007981.s004.pdf]

Medellín, November 5th, 2019

Response to reviewers

Reviewer #1:

The objective of the study is clearly stated and the study design is well described. However, the statistical methods used are not fully described. For example, what statistical method was used to test differences in the accuracies.

Reply: A covariate was assumed to have a significant effect on the estimates of sensitivity and specificity and thus to explain some of the heterogeneity in the studies included in meta-analysis if the P value was 0.05 using mixed-effects logistic regression (please see lines 209-211).

Tables 1 and 2 are not available for review.

Reply: We are sorry. Table files were submitted with other files

Line 235-236: There is no pooled estimate in Figure 2.

Reply: There was a mistake. Text was corrected. "A coupled forest plot of sensitivity and specificity of PCR are shown in Fig. 3" (see lines 267-268)

Line 239-241: The authors wrote here and other places that "there were no significant differences in the accuracies between various methods." What statistical analysis was used to test these difference.

Reply: Statistical analysis was based on Chi-square. The following text was included: Diagnostic accuracies between various readout methods for PCR (index tests), PCR samples, the study design and reference standard, were compared using Chi-square test with the programme STATA version 14. (See lines 228-230)

Line 278-279: Revise the statement.

Reply: the sentence was revised. In this systematic review, we analyzed and summarized data from diagnostic accuracy studies of molecular test in the diagnosis of CL (See lines 313-314).

Line 287: Do you mean: There was, however, "no significant" or "a non-significant" difference...

Reply: Differences were no significant. This sentence was corrected in text

Although the results in this study are well discussed, the author almost failed to provide justifications for their findings. What are the implications for low accuracies of these tests? What are the implications?

Reply: Implications were included in Discussion section

Writes confidence interval (CI) in full only the first time used and use CI subsequently.

Reply: CI was used subsequently

2. May I suggest the authors separate "Methods" and "Results"

Reply: This was revised and corrected along text.

3. Page 3 line 74-75: Provide reference for this statement "The evolution time of the lesion and the previous use of treatments may also interfere with the sensitivity of the direct methods."

Reply: a reference was included. See reference #1

line 115: Remove the inverted question mark

Reply: Question mark was deleted

line 152: I was wondering why the authors removed all studies where a 2 by 2 contingency table cannot be created?

Reply: It was established as inclusion criteria that the studies to have the ability to complete a 2x2 contingency table because these data are necessary to calculate the diagnostics accuracies of the tests, additionally poor quality of reporting generate biases in diagnostic accuracy test studies (STARD standards).(See lines 158-164).

Line 211: You mean "<0.05"?

Reply: Yes. We mean < 0.05. This was corrected in text.

Reviewer #2:

1. For heterogeneity analysis gold standard test were a. direct microscopy and b. microscopy and or culture. However, for generation of HSROC authors have again used gold standard A and B and here it indicates the sample type A. smear B. invasive sample. For clarity and comparative representation fig 3 and fig 4 can be merged in to one as 3 a and 3b and it may be indicated that this analysis is about the type of sampling procedure used. As it is one of the main findings and should be emphasized from patients point of view highlighting the use of non-invasive sampling procedure.

Reply: *Graph 3 and 4 were merge as 4a and 4b.*

In order to achieve accurate review results, we used a first subgroup analysis that included studies that used exclusively the direct microscopic test as a gold standard, A second subgroup where the gold standard included both direct microscopic test and/or culture but with comparable or similar methodologies (Please see lines 198-202).

2. Line 300-302, it may be rephrased to give clear directions for the future diagnostic accuracy studies to be carried out, instead of stating that future studies will support the findings of this study.

Reply: *Regarding to the future diagnostic accuracy studies, we highlight the fact of leave out the case-control studies to avoid that diagnostic accuracy being overestimated and considered the results for PCR in simple smears sample of this systematic review and meta-analysis such as the most accurate method for the diagnosis of CL instead more invasive samples [24] (Please see lines 313-317).*

3. The language is not lucid at several places and interpretation become difficult, it may be improved. eg line 264; 278; 287

Reply: These text were added:

In 10 out of 13 studies, the patient's flow could have introduced bias; there was no details about that the results of the index test and reference standard were collected on the same patients at the same time (Please see lines 271-273).

The summary estimates for sensitivity and specificity for both "All readout methods of the index test except LAMP and Real-time PCR" and "All readout methods of the index test without exception" on smears samples (general population) were high; however, estimates were lower on aspirate, skin biopsies and swab samples in general population; there was no statistically significant difference between the diagnostic accuracy in smears, aspirate, skin biopsies or swab samples. This finding allows us to believe that a simple smears sample would suffice instead of taking more invasive skin biopsies or aspirate samples.

This finding is important from patient's point of view because highlighting the use of non-invasive sampling procedures (please see lines 284-292).

This is to be expected, because the controls in case-control studies are often healthy persons, whereas controls in consecutive studies are in fact suspected patients; variation in the controls were one of the most frequent points of heterogeneity among the studies [7]. Please see lines 297-300).

Reviewer #3:

-Are the objectives of the study clearly articulated with a clear testable hypothesis stated? No

Reply: The last paragraph in the Introduction section was modified: *Providing clear evidence on the diagnostic accuracy of molecular tests allows to clarify the role of molecular techniques in epidemiological contexts. Here the importance of knowing the diagnostic accuracy of molecular tests so that public health institutions and those in charge of making decisions may implement the use of theses test for the control of CL. Based on the hypothesis that the PCR-based molecular tools are the most accurate diagnostic method, the objective of this systematic review was to assess the diagnostic attributes of PCR-based molecular tools in a meta-analysis of the published literature, with the purpose of contribute to the improvement of CL control. Please see lines 105-112).*

This hypothesis was included in text (please see lines

-Is the study design appropriate to address the stated objectives? Yes

Reply: not applicable

-Is the population clearly described and appropriate for the hypothesis being tested? Yes

Reply: not applicable

-Is the sample size sufficient to ensure adequate power to address the hypothesis being tested? Not applicable

Reply: not applicable

-Were correct statistical analysis used to support conclusions? Yes

Reply: not applicable

-Are there concerns about ethical or regulatory requirements being met? No

Reply: not applicable

Are the conclusions supported by the data presented?: No conclusions in the main manuscript!

Reply: A Conclusion section was included in the manuscript. See lines 355-361

This paper systematically reviews and meta-analyses the sensitivity and specificity of PCR-based tests when compared to direct microscopy (+/- isolation of promastigotes in cultures) at diagnosing cutaneous leishmaniasis. It is generally sound in its methods but the writing needs more work in my opinion. Here are some comments/suggestions:

The objective and the primary conclusion don't align. This should be corrected.

Reply: This was corrected. See lines 22-27.

Introduction:
Perhaps the author could consider shortening it, and should clearly describe the concepts of the clinical workflow in the target condition, prior tests (like clinical suspicion), index test and comparator tests, and intended role of the index test in the clinical workflow.

Quizás el autor podría considerar acortarlo, y debería describir claramente los conceptos del flujo de trabajo clínico en la condición objetivo, pruebas previas (como sospecha clínica), prueba de índice y pruebas de comparación, y el papel previsto de la prueba de índice en el flujo de trabajo clínico.

Reply: Thanks for the comment. However, we consider that the Introduction is adequate

No objective statement in the introduction section.

*Reply: The statement with the objectives was included in the introduction section *see lines 111-113).*

Materials and methods

Why wasn't PRISMA-DTA not followed? This is the usual reporting standard for diagnostic systematic reviews

Reply: *The PRISMA–DTA file was not available in the web page of Plos NTD at the momment when the manuscript was submmited. In the revised version PRISMA file was replaced by PRISMA-DTA.*

Was any grey literature source searched? Were conference abstracts excluded?

Reply: We decided no include any source of gray literature and conference abstracts. Only literature published in the databases of greater medical coverage were included, because in this way we considered that we covered the most important findings reported on the diagnosis of CL.

Search terms: I would argue including concepts of direct tests and humans restricts the search term unnecessarily ("direct exam" OR "direct test" OR "direct tests" OR "direct microscopy" OR smear)) AND Humans. How many studies were lost due to inclusion of these terms in the search term with Boolean concept AND? How many of them were relevant?

Reply: *The search included all the concepts with which the reference test could be named in the medical literature. This was done in order to include all the articles that named this test in different ways. The "human" concept was included to refine the search and leave out the diagnostic studies of LC in animals, which were considerable. It is important to clarify that of the different search options that were carried out, this was the one that was considered suitable. The exact quantities of articles found in the different searches carried out were not registered, since they yielded many useless references for our purpose.*

Analysis of the heterogeneity of the papers: suggest also considering the inclusion of treated patients and study location as sources of heterogeneity between studies.

Reply: Patients who received prior treatment for CL may interfere with an accurate diagnosis, therefore they were excluded from diagnostic accuracy studies in CL. The study location was not considered an important source of heterogeneity to assess in CL in this work.

"Heterogeneity was assessed using Cochran's Q test as well as Higgins' I2 statistic": no results eventually provided. I would suggest excluding them altogether

Reply: It was removed

Study inclusion: a number of studies have double counting of patients. Authors should be transparent about this as this means that this results in a faulty over-assumption about the variability by the statistical models.

Reply: Although there was a patinet re-counts, the Table 1 adequately details the origin of each data, and this is clarified in the text (See lines 157-158).

reconsider denoting direct test as the “gold standard”. It isn’t, indeed PCR based methods are generally thought to have better accuracy. Instead, consider using “reference standard”

Reply: This was corrected along the text.

“Statistical analysis and meta-analysis.”: sounds odd together. Suggest just use either “Statistical analysis” or “meta-analysis”.

Reply: The meta-analysis was deleted. (See line 216)

Suggest mentioning the model before the covariates.

Reply: This was corrected. (see line 219)

Were the covariates entered together or individually? Since this was the formal way that the authors tested for heterogeneity, in my opinion, the former section should be called “sources of heterogeneity” and the actual assessment of heterogeneity detailed under statistical analysis.

Reply: The covariates were entered through the method forward stepwise.

“heterogeneity in the studies included in meta-analysis if the P value was 0.05…”: authors do not mention any P value in the results.

Reply: P values were included in the text.

while there is no recommended method to test for publication bias, a statement regarding this should be included. [I would also suggest considering inspection of funnel plot (diagnostic odds ratio vs sample size) and including this as a post-hoc analysis in supplementary material. I understand no inferences can be made regarding the presence or absence of publication bias though.]

Reply: Since there is no recommended method to assess the publication bias and that no inferences can be made regarding the presence or absence of this bias; in this systematic review and meta-analysis was not assessed the publication bias, although we recognize that this bias is a serious problem, which can affect the validity and generalization of conclusions.

“Study characteristics” usually connotes study design. Instead authors list PCR methods and source of biological material. Consider reorganizing.

Reply: They were reorganized. The title “Study characteristics” was deleted and included in the prior section “Included studies”.

“Quality assessment of study reports” logically comes before the main results (and so perhaps should the associated figures). Consider reorganizing

Reply: This was reorganized. “Quality assessment of study reports” was located before “Diagnostic accuracy of molecular tests and analysis of heterogeneity”

Diagnostic accuracy of molecular tests and analysis of heterogeneity: consider adopting a more narrative style rather than just pointing out tables and figures.

Reply: The text was revised

Table 2, as presented, is very confusing. If it represents subgroup analyses, then one would intuitively think that the subgroups would be consecutively recruited vs Case-control (indeed, as stated in the analysis section), and not the combined categories.

Reply: Given the poor quality of the report presented by the diagnostic accuracy studies and the lack of homogeneity of them, it is very complex to find the appropriate way to group the main findings. For this reason, we found that presenting them grouped in the two most frequently types of studies reported in the literature for diagnostic accuracy tests, and within these two large groups of studies, including the main characteristics analyzed, is the ideal form in our case, of showing the results.

Figure 1. Consider detailing the “inappropriate gold standard”s since 15 studies were excluded because of this criterion.

Reply: A footnote was included in the Figure 1

Why is PRISMA flow chart also included as supplementary material?

Reply: The old Fig 1 was replaced by PRISMA flow chart.

Discussion:

Generally poorly organized.

Reply: Discussion section was carefully revised and modified according to comments

Page 10, 283-290: these are results.

Reply: They were moved to Results

Page 10, 289-290: is this analysis post-hoc? it is not mentioned in the methods

Reply: This was a mistake and sentence was deleted.

No conclusion

Reply: Conclusions were included in the manuscript

Other stylistic issues:

Abstract:
Background: "….PubMed, EMBASE and LILACS": this should be part of the Methodology

Reply: This was corrected

Methodology/Principal findings:
Suggest excluding "This is to be expected, because the controls in case-control studies are often healthy persons, whereas controls in consecutive studies are in fact suspected patients."

Reply: This was corrected

The following sentence is grammatically incorrect: "Estimates were lower on aspirates, skin biopsies and swab samples in general population 0.90 (95% confidence interval [CI], 0.80 to 0.95) and 0.87 (95% confidence interval [CI], 0.76 to 0.94) for sensitivity and specificity, respectively."

Reply: This was corrected

"More than 30 Leishmania species are recognized, of which 20 are considered infective for humans and other mammals." This sentence should be referenced.

Reply: A reference was included See reference #1

Other examples of grammatical errors or stylistic concerns include but not limited to:

" significant differences between the readout method of the index test, however, subgroups were analyzed separately: "all readout methods of the index test except LAMP and real-time PCR" and "all readout methods of the index test without exception".

Reply: This was corrected

“The lack of standardization is another limitation when you want to compare diagnostic accuracy studies for molecular tools.”

Reply: This was corrected

Page 3, 70-71; Page 4, 95; Page 7, 196-198; Page 9, 264-265

Reply: These texts were revised
