# Supplementary material for: Test accuracy of polymerase chain reaction methods against conventional diagnostic techniques for Cutaneous Leishmaniasis (CL) in patients with clinical or epidemiological suspicion of CL: Systematic review and meta-analysis
Source: PLoS Negl Trop Dis. 2020 Jan 21;14(1):e0007981. doi: 10.1371/journal.pntd.0007981 (PMC6994169; doi:10.1371/journal.pntd.0007981)
Supplement: Attachment — Submitted filename: Review PNTD CL metaanalysis.docx [file pntd.0007981.s003.docx]

The manuscript by Mesa et al presents a systematic review and meta-analysis regarding the test accuracy of polymerase chain reaction methods against conventional diagnostic

techniques for Cutaneous Leishmaniasis (CL) in patients with clinical or

epidemiological suspicion of CL. The authors have used both case control and consecutive studies for this purpose as the studies pertaining to this are very limited and pooled analysis of all the molecular diagnostic methods including PCR, LAMP, RPA, real time PCR were carried out.

The review protocol adopted for analysis followed by authors is standard. In conclusion from 12/13 studies from which the data could be extracted the conclusion was drawn that the molecular methods have high sensitivity and specificity in all the readout methods, and the accuracy of test is similar in smears, aspirate, skin biopsies or swabs samples. In conclusion, authors recommend that a simple smears sample run by PCR instead more invasive samples is enough to obtain a positive diagnosis of CL.

Comments:

1. For heterogeneity analysis gold standard test were a. direct microscopy and b. microscopy and or culture. However, for generation of HSROC authors have again used gold standard A and B and here it indicates the sample type A. smear B. invasive sample.

For clarity and comparative representation fig 3 and fig 4 can be merged in to one as 3 a and 3b and it may be indicated that this analysis is about the type of sampling procedure used. As it is one of the main findings and should be emphasized from patients point of view highlighting the use of non invasive sampling procedure.

2. Line 300-302, it may be rephrased to give clear directions for the future diagnostic accuracy studies to be carried out, instead of stating that future studies will support the findings of this study.
3. The language is not lucid at several places and interpretation become difficult, it may be improved. eg line 264; 278; 287
